# Lysine Acetylation Regulates Alanyl-tRNA Synthetase Activity in *Escherichia coli*

**DOI:** 10.3390/genes9100473

**Published:** 2018-09-28

**Authors:** Takuya Umehara, Saori Kosono, Dieter Söll, Koji Tamura

**Affiliations:** 1 Biotechnology Research Center, The University of Tokyo, Tokyo 113-8657, Japan; uskos@mail.ecc.u-tokyo.ac.jp; 2 Department of Biological Science and Technology, Tokyo University of Science, Tokyo 125-8585, Japan; koji@rs.tus.ac.jp; 3 Center for Sustainable Resource Science, RIKEN, Saitama 351-0198, Japan; 4 Department of Molecular Biophysics and Biochemistry, Yale University, New Haven, CT 06520, USA; dieter.soll@yale.edu; 5 Department of Chemistry, Yale University, New Haven, CT 06520, USA; 6 Research Institute for Science and Technology, Tokyo University of Science, Chiba 278-8510, Japan

**Keywords:** alanyl-tRNA synthetase, class II aminoacyl-tRNA synthetase, expanded genetic code, lysine acetylation, posttranslational modification

## Abstract

Protein lysine acetylation is a widely conserved posttranslational modification in all three domains of life. Lysine acetylation frequently occurs in aminoacyl-tRNA synthetases (aaRSs) from many organisms. In this study, we determined the impact of the naturally occurring acetylation at lysine-73 (K73) in *Escherichia coli* class II alanyl-tRNA synthetase (AlaRS) on its alanylation activity. We prepared an AlaRS K73Ac variant in which *N^ε^*-acetyl-l-lysine was incorporated at position 73 using an expanded genetic code system in *E. coli*. The AlaRS K73Ac variant showed low activity compared to the AlaRS wild type (WT). Nicotinamide treatment or CobB-deletion in an *E. coli* led to elevated acetylation levels of AlaRS K73Ac and strongly reduced alanylation activities. We assumed that alanylation by AlaRS is affected by K73 acetylation, and the modification is sensitive to CobB deacetylase in vivo. We also showed that *E. coli* expresses two CobB isoforms (CobB-L and CobB-S) in vivo. CobB-S displayed the deacetylase activity of the AlaRS K73Ac variant in vitro. Our results imply a potential regulatory role for lysine acetylation in controlling the activity of aaRSs and protein synthesis.

## 1. Introduction

The recent development of mass spectrometry (MS)-based proteomic analysis enables the discovery of a wide variety of posttranslational modifications (PTMs) in prokaryotes [1]. Acetylation of lysine residues in proteins is distributed across all domains of life and is thought to be functionally important. Proteomic studies of acetylated protein (acetylome) indicated a large number of acetylated proteins in bacteria and archaea [2,3,4,5,6,7]. Since they also possess homologs of eukaryotic lysine acetyltransferases (KATs) and deacetylases (KDACs) [8], acetylation in bacteria and archaea are expected to be involved in the regulation of cellular processes as in eukaryotes. Recently, there has been growing interest in the role of acetylation [9,10,11,12,13,14]; however, the regulatory mechanisms and functional implications of prokaryotic acetylation are still largely unknown.

In *Escherichia coli*, acetylation is introduced to lysine residues by a KAT-dependent reaction utilizing acetyl-CoA and a non-enzymatic reaction utilizing acetyl-phosphate [15,16]. The non-enzymatic system is involved in global protein acetylation under carbon stress [17]. Moreover, the sirtuin-type KDAC CobB is responsible for deacetylation of many proteins [18]. Acetylome analyses in *E. coli* have identified hundreds of acetylated proteins [7,15,16,17,19,20]; the most abundant group was metabolism-related proteins followed by translation-related proteins. Many of the translation-related proteins interact with RNA and nucleotides for their function. As the positive charge of lysine residues in those proteins is often crucial for their interaction with RNA and nucleotides, acetylation that cancels the charged lysine may affect function. We therefore searched for lysine acetylation found in aminoacyl-tRNA synthetases (aaRSs) (Table A1), which are essential enzymes to synthesize aminoacyl-tRNA in the first step of protein translation. Acetylation has been identified in all species of aaRSs and most of the acetylation sites seem to be located away from the catalytic and substrate-binding sites. However, some acetylations were detected at lysine residues, which are important for their aminoacylation activity and are expected to control the reaction.

Recently, it has been shown that naturally occurring acetylation of lysine residues in class I aaRSs, leucyl-tRNA synthetase (LeuRS), arginyl-tRNA synthetase (ArgRS), and tyrosyl-tRNA synthetase (TyrRS) in *E. coli* downregulates their enzymatic activities by using the expanded genetic code system with *N^ε^*-acetyl-l-lysine (AcK). It was demonstrated that acetylation of K619 and K624 in LeuRS, which are located within and next to the conserved KMSKS motif involved in ATP recognition [21], and acetylation of K809, which interacts with leucine tRNA, impaired enzymatic activity [22]. They further elucidated that acetylation of K126 and K408 in ArgRS, which are involved in ATP binding and interaction with arginine tRNA, respectively, also decreases the enzymatic activity. Acetylation of K85, K235 and K238 in TyrRS reduced the enzyme activity; K235 and K238 are conserved lysine residues of the KMSKS motif and form the catalytic domain with K85 [23]. All of the acetylations in the three aaRSs are sensitive to CobB.

Although the impact of lysine acetylation on the class I aaRSs has been shown, the impact on class II enzymes, which have structurally different catalytic domains from those of class I [21], has not been examined. In this study, we demonstrate that one of the class II aaRSs, alanyl-tRNA synthetase (AlaRS) from *E. coli*, is regulated by acetylation at lysine-73 (K73). We further provide the first evidence that *E. coli* expresses two CobB isoforms in vivo and that its short variant (CobB-S) has deacetylase activity for K73 of AlaRS.

## 2. Materials and Methods

### 2.1. General

Custom oligonucleotide synthesis was ordered from Eurofins Genomics (Tokyo, Japan). The primer sequences used in this study are described in Table A2. DNA sequencing was performed by an ABI 3130xl (Applied Biosystems, Foster City, CA, USA) in the Research Equipment Center, Tokyo University of Science. Commercially available chemicals and enzymes were purchased from Wako Pure Chemical (Osaka, Japan) and Takara Bio (Shiga, Japan), respectively, unless otherwise noted. The plasmids and *E. coli* strains used in this study are listed in Table A3 and Table A4, respectively.

### 2.2. Plasmid Constructions

**Figure A1:**
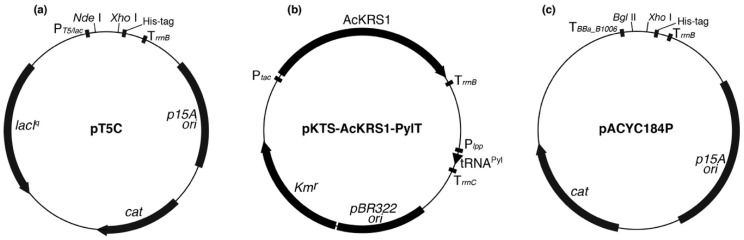
Plasmid maps of (**a**) pT5C, (**b**) pKTS-AcKRS1-PylT, and (**c**) pACYC184P. P*_T5/lac_*, P*_lpp_*, and P*_tac_* indicate promoter. T*_rrnB_* and T*_rrnC_*, and T*_BBa_B1006_* indicate transcription terminator. The artificial terminator BBa_B1006 blocks intrinsic promoter activity.

The pT5C plasmid originated from pACYC184, in which a tetracycline resistant gene (*tet*^R^) was replaced by the T5/*lac* promoter, C-terminal His-tagged sequence, the *rrnB* terminator, and the *lacI*^q^ gene (Figure A1a). The AlaRS gene (*alaS;* ACB03816) from *E. coli* DH10B (Invitrogen, Carlsbad, CA, USA) was inserted between the *Nde*I and *Xho*I site in pT5C, resulting in pT5C-Ec-alaS. The lysine codon (AAA) at position 74 of the *alaS* gene on pT5C-Ec-alaS was substituted to an alanine (GCG) or amber stop (TAG) codon by site-directed mutagenesis, resulting in pT5C-Ec-alaS-K74A and pT5C-Ec-alaS-K74amb, respectively. The discrepancy between K73 and the codon number is due to the posttranslational removal of the first methionine (Met) in cells [24].

pKTS-AcKRS1-PylT (Figure A1b), which was used for genetic incorporation of AcK, was constructed by inserting a *Methanosarcina mazei* pyrrolysine tRNA (tRNA^Pyl^) expression cassette from pTECH-PylT into the *Nhe*I site of pKTS-AcKRS1, which overexpresses an *N^ε^*-acetyl lysyl-tRNA synthetase (AcKRS) in *E. coli* [25].

For expression and promoter analyses, the pACYC184P was constructed from pACYC184, in which *tet*^R^ was replaced by the artificial transcription terminator (BBa_B1006), C-terminal His-tagged sequence, and the *rrnB* terminator (Figure A1c). CobB open reading frames (ORFs) (*cobB* gene; ACB02313) with the 1218, 417, and 30 nucleotide upstream sequences, *cobB*-*L* ORF, and *cobB*-*S* ORF (starts from the second Met codon of *cobB*-*L* ORF; Figure 5b) were inserted between the *Bgl*II and *Xho*I site in pACYC184P, resulting in pACYC184P-1218-cobB, pACYC184P-417-cobB, pACYC184P-30-cobB, pACYC184P-cobB-L, and pACYC184P-cobB-S.

For purification of the N-terminal His-tagged CobB protein, *cobB*-*L* and *cobB*-*S* ORFs were inserted between the *Nde*I and *Bam*HI site of pET15b (Novagen, Madison, WI, USA), resulting in pET15b-cobB-L and pET15b-cobB-S, respectively.

### 2.3. Construction of CobB-Deleted Escherichia coli Strain

To construct the CobB-deleted *E. coli* (∆CobB) strain, the *cobB* gene was removed from the *E. coli* DH10B genome by using the λ-red recombination system [26,27]. The kanamycin resistant gene was next removed by FLP–FRT recombination. The gene deletion was verified by PCR.

### 2.4. Protein Expression and Purification

For preparation of the C-terminal His-tagged AlaRS wild type (WT) and its K73A mutant, *E. coli* DH10B transformed with pT5C-Ec-alaS or pT5C-Ec-alaS-K74A were grown in Luria-Bertani (LB) medium (1% Trypton, 0.5% yeast extract, and 1% NaCl) supplemented with 34 µg/mL chloramphenicol at 37 °C. When the OD_600_ of the culture reached 0.6, Isopropyl-β-D-1-thiogalactopyranoside (IPTG) was added to a final concentration of 0.2 mM and the cultivation was continued for 4 h. The cell pellet was resuspended with lysis buffer (20 mM Tris-HCl pH 8.0, 300 mM NaCl, and 10 mM imidazole) supplemented with 0.2 mg/mL lysozyme and 0.2% Triton X-100, and broken by sonication. After centrifugation (13,000× *g*, 4 °C, 20 min) to remove cell debris, the supernatant was charged onto a Ni-NTA agarose (Qiagen, Hilden, Germany) column equilibrated with lysis buffer. The column was washed with wash buffer (20 mM Tris-HCl pH 8.0, 300 mM NaCl, and 50 mM imidazole), and His-tagged AlaRS was eluted with elution buffer (20 mM Tris-HCl pH 8.0, 300 mM NaCl, and 250 mM imidazole). Fractions containing a homogeneous protein enzyme were pooled and dialyzed twice in 1 L of dialysis buffer (20 mM Tris-HCl pH 8.0, 10 mM MgCl_2_, and 50 mM KCl) followed by concentration by Amicon Ultra Ultracel-30K (Merck Millipore, Billerica, MA, USA). Finally, the enzymes were stored in 10 mM Tris-HCl pH 8.0, 5 mM MgCl_2_, 25 mM KCl, 2 mM dithiothreitol (DTT), and 50% glycerol.

For preparation of C-terminal His-tagged AcK-incorporated AlaRS (AlaRS K73Ac), *E. coli* DH10B or its ∆CobB strain co-transformed with pKTS-AcKRS1-PylT and pT5C-Ec-alaS-K74amb were grown in LB medium supplemented with 25 µg/mL kanamycin and 34 µg/mL chloramphenicol at 37 °C. Twenty millimolar of nicotinamide (NAM; Sigma-Aldrich, St. Louis, MO, USA), which is an inhibitor of CobB deacetylase, was added if necessary. When the OD_600_ of the culture reached 0.5, IPTG and AcK (Sigma-Aldrich) were added to a final concentration of 0.2 mM and 2 mM, respectively, and the cultivation was continued for 8 h. His-tag affinity purification was performed as described above.

For preparation of N-terminal His-tagged CobB protein used for the deacetylation assay, *E. coli* BL21(DE3) (Nippon Gene, Tokyo, Japan) transformed with pET15b-cobB-S was grown in LB medium supplemented with 100 µg/mL ampicillin at 37 °C. When the OD_600_ of the culture reached 0.6, IPTG was added to a final concentration of 0.2 mM. The cells were continuously cultivated for 4 h and then harvested. His-tag affinity purification was performed as described above. The purified enzyme was dialyzed in 1 L of dialysis buffer (20 mM Tris-HCl pH 8.0 and 100 mM NaCl) twice and concentrated by Amicon Ultra Ultracel-10K (Merck Millipore). Finally, the enzyme was stored in 10 mM Tris-HCl pH 8.0, 50 mM NaCl, 2 mM DTT, and 50% glycerol.

### 2.5. Western Blotting

After SDS-PAGE, proteins were electroblotted onto a Hybond-ECL membrane (GE Healthcare, Buckinghamshire, UK), which was blocked in Tris-buffered saline containing 0.1% Tween-20 (TBS-T) and 5% skimmed milk. Proteins on the membrane were probed with antibody in blocking buffer and then the membrane was washed with TBS-T. Rabbit polyclonal anti-AcK antibody (ImmuneChem, Burnaby, BC, Canada) and Anti-His-tag mAb-HRP-DirecT (Medical & Biological Laboratories, Aichi, Japan) were used at a 1/3000 and 1/10,000 dilution respectively as primary antibodies. Anti-Rabbit IgG HRP conjugate (Promega, Madison, WI, USA) was used as the secondary antibody. Proteins on the membrane were probed with the primary antibody in blocking buffer and then washed with TBS-T. Proteins were detected by Immobilon Western Chemilum HRP substrate (Merck Millipore) and visualized on a LAS 4000 (GE Healthcare).

### 2.6. Alanylation Assay

Alanine tRNA (tRNA^Ala^) from *E. coli* was transcribed in vitro by T7 RNA polymerase and purified by 12% polyacrylamide gel with 7 M urea. The alanylation assay was performed at 37 °C in a reaction mixture (50 µL) containing 50 mM HEPES-NaOH pH 7.4, 10 mM MgCl_2_, 30 mM KCl, 2 mM ATP, 10 µM [U-^14^C] Ala (164 mCi/mmol; GE Healthcare), 5 µM transcribed tRNA^Ala^, and 5 ng/µL (50 nM) His-tagged AlaRS or its variants. At time points of 2 min, 4 min, 8 min, and 10 min, 10 µL aliquots of the reaction mixture were spotted onto Whatman 3MM filter paper (GE Healthcare) and the reaction was immediately quenched with 5% trichloroacetic acid (TCA). After washing the filter paper with 5% TCA and drying, radioactivity on the filter paper was counted by a Beckman LS 6500 Scintillation Counter (Beckman Coulter, Brea, CA, USA).

### 2.7. Circular Dichroism Spectrometry Analysis

The circular dichroism (CD) spectra of AlaRS and its derivatives were recorded on a J-805 CD Spectrometer (JASCO Corporation, Tokyo, Japan). AlaRS WT, K73A, and K73Ac (NAM+) were respectively prepared at a concentration of 0.1 mg/mL (1.0 µM) in a buffer containing 5 mM Tris-HCl pH 7.5 and 5 mM MgCl_2_, and scanned from 195 nm to 265 nm with a 50 nm/min speed.

### 2.8. Expression and Promoter Analysis for CobB-L and CobB-S

*E. coli* DH10B cells harboring pACYC184P-1218-cobB, pACYC184P-417-cobB, pACYC184P-30-cobB, pACYC184P-cobB-L, or pACYC184P-cobB-S were cultivated in LB medium at 37 °C overnight. The cells were broken in lysis buffer (50 mM Tris-HCl pH 8.0, 100 mM NaCl, 1 mM EDTA, 2% Triton X-100, 1% SDS, and 10% glycerol) by sonication. The protein concentration of the lysates was determined by an XL-Bradford (APRO SCIENCE, Tokushima, Japan). Ten micrograms of protein were subjected to 15% SDS-PAGE and Western blotting analysis using Anti-His-tag mAb-HRP-DirecT to detect the His-tagged CobB expression.

### 2.9. Deacetylation Assay

The assay was performed at 37 °C for 12 h in a reaction mixture (30 µL) containing 25 mM Tris-HCl pH 8.0, 100 mM NaCl, 27 mM KCl, 0.5 mM NAD^+^ (Nacalai, Kyoto, Japan), 0.5 µM AlaRS K73Ac (∆CobB), and 1.5 or 5 µM His-tagged CobB-S. The reaction was stopped by adding an equal volume of 2× sample buffer for SDS-PAGE. The resulting samples containing 0.2 pmol of AlaRS were separated by 7.5% SDS-PAGE and acetylation levels of the AlaRS K73Ac (∆CobB) were determined by Western blotting analysis using anti-AcK antibody.

## 3. Results

### 3.1. Preparation of AlaRS K73Ac Variant by Expanded Genetic Code

**Figure 1:**
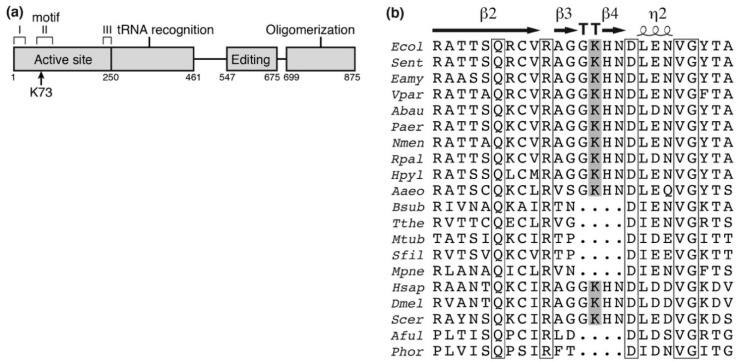
Schematic position of lysine-73 (K73) acetylation site in alanyl-tRNA synthetase (AlaRS). (**a**) Schematic representation of AlaRS with functional domains. K73 is located within the motif II, an essential constituent for the active site. (**b**) The alignment of sequences surrounding K73 in AlaRS. Parentheses indicate accession number. *Ecol*, *Escherichia coli* (ACB03816); *Sent*, *Salmonella enterica* (WP_065680553); *Eamy*, *Erwinia amylovora* (WP_004155915); *Vpar*, *Vibrio parahaemolyticus* (WP_086591229); *Abau*, *Acinetobacter baumannii* (ACC56466); *Paer*, *Pseudomonas aeruginosa* (WP_034012995); *Nmen*, *Neisseria meningitidis* (WP_010980961); *Rpal*, *Rhodopseudomonas palustris* (WP_044410751); *Hpyl*, *Helicobacter pylori* (WP_000354743); *Aaeo*, *Aquifex aeolicus* (WP_010880825); *Bsub*, *Bacillus subtilis* (WP_086344136); *Tthe*, *Thermus thermophilus* (WP_011228945); *Mtub*, *Mycobacterium tuberculosis* (WP_055366958); *Sfil*, *Streptomyces filamentosus* (WP_006123197); *Mpne*, *Mycoplasma pneumoniae* (WP_014574975); *Hsap*, *Homo sapiens* (NP_001596); *Dmel*, *Drosophila melanogaster* (NP_523511); *Scer*, *Saccharomyces cerevisiae* (EDV10897); *Aful*, *Archaeoglobus fulgidus* (WP_010879744); *Phor*, *Pyrococcus horikoshii* (WP_010884393); whole sequences were aligned by ClustalW [32] and ESPript 3 [33]. K73 in *E. coli* AlaRS is shown with the gray box and lies on the loop in the β-turn (TT). Strictly conserved residues among all organisms are shown with open boxes.

The previously reported *E*. *coli* acetylome studies identified 15 lysine acetylation sites in AlaRS (Table A1). Of them, K73 is an essential residue in motif II of the enzyme active site (Figure 1a) [28] and interacts with the 3′-end of the cognate tRNA^Ala^ [29,30]. In addition, this residue is well-conserved among several bacteria and eukaryotes (Figure 1b). We therefore investigated the impact of acetylation at K73 of AlaRS using the genetic incorporation of AcK into position 73 of AlaRS. The expanded genetic code system is a technology to reassign non-canonical amino acids to one of the stop codons (usually the amber codon) by pairs of engineered aaRS and its cognate suppressor tRNA [31]. One such pair, AcKRS/tRNA^Pyl^ from *M*. *mazei*, can co-translationally introduce AcK at the amber codon, resulting in production of a protein with AcK at the specified sites [25]. Since the protein homogenously prepared by this system contains AcK, the protein with AcK is suitable to examine the impact of lysine acetylation.

**Figure 2:**
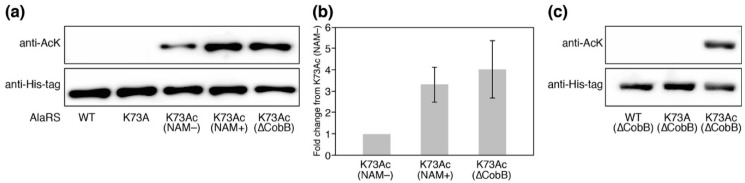
The verification of K73Ac-incorporation in AlaRS by Western blotting. (**a**) The acetylation levels of AlaRS variants were detected by Western blotting analysis using an anti- *N^ε^*-acetyl-l-lysine (AcK) antibody. Western blotting analysis using an anti-His-tag antibody showed the loaded protein control. NAM indicates nicotinamide. The data is representative of three independent SDS-PAGE and Western blotting assays. (**b**) Quantification of the band intensities of three independent Western blotting assays. The relative acetylation levels of K73Ac (NAM+) and K73Ac (∆CobB) to that of K73Ac (NAM−) were calculated. (**c**) Acetylation levels of the AlaRS wild-type (WT) and K73A mutant that were expressed under the same conditions as the K73Ac (∆CobB) variant.

**Figure A2:**
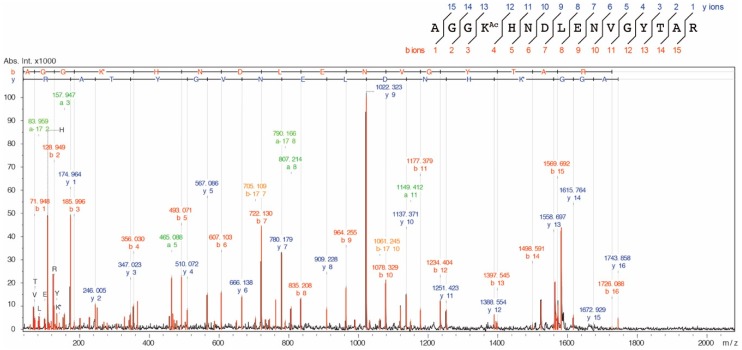
MS/MS ion spectrum of the K73Ac-acetylated peptide (71-AGGK^Ac^HNDLENVGYTAR-86, *m/z* 1743.839). AlaRS K73Ac (∆CobB) was reduced with 20 mM dithiothreitol (DTT) and subsequently alkylated with 30 mM iodoacetamide and digested with a sequence-grade trypsin (Promega). The proteolytic peptides were cleaned using a ZipTip C18 column (Merck Millipore) and analyzed by an autoflex speed TOF/TOF mass spectrometer (Bruker, Billerica, MA, USA).

We prepared the AlaRS K73Ac variant using the AcKRS/tRNA^Pyl^ system and verified AcK-incorporation in AlaRS by Western blotting with the anti-AcK antibody (Figure 2a). Specific incorporation of AcK into position 73 of AlaRS was also verified by MALDI-TOF/TOF analysis (Figure A2). It has been reported that CobB is responsible for deacetylation against many proteins in *E. coli* [18]. In order to know whether acetylation of K73 was reversed by CobB, we examined the acetylation levels of AlaRS K73Ac purified from NAM-treated (NAM+) and untreated (NAM−) *E. coli* DH10B. NAM is an inhibitor of sirtuin-type lysine deacetylases including CobB. AlaRS K73Ac (NAM+) showed higher acetylation signals than AlaRS K73Ac (NAM−), although AlaRS WT and its K73A mutant as negative controls did not give a signal of acetylation. The observation that acetylation of the AlaRS WT and K73A mutant could not be detected by Western blotting is thought to be due to the amount of the overexpressed enzymes being excess to a capability of endogenous lysine acetylation, resulting in reducing populations of acetylated AlaRS. We next examined the acetylation level of K73Ac purified from the ∆CobB strain. The acetylation level of K73Ac (∆CobB) was almost the same as that of K73Ac (NAM+). Quantification of the band intensity showed that the acetylation levels of K73Ac (NAM+) and K73Ac (∆CobB) were 3.3-fold and 4.0-fold higher than that of K73Ac (NAM−), respectively (Figure 2b). These results indicated that NAM treatment and CobB deletion enhanced the acetylation of AlaRS, suggesting that CobB can deacetylate K73Ac in vivo. It should be noted that lysine acetylation was not detected in the AlaRS WT and K73A mutant purified from the ∆CobB strain (Figure 2c). Taken together, these results support that the acetylation signal of the Western blot represented the K73 acetylation and CobB was responsible for the decreased acetylation of K73Ac.

### 3.2. Alanylation Activity of AlaRS K73Ac Variants

**Figure 3:**
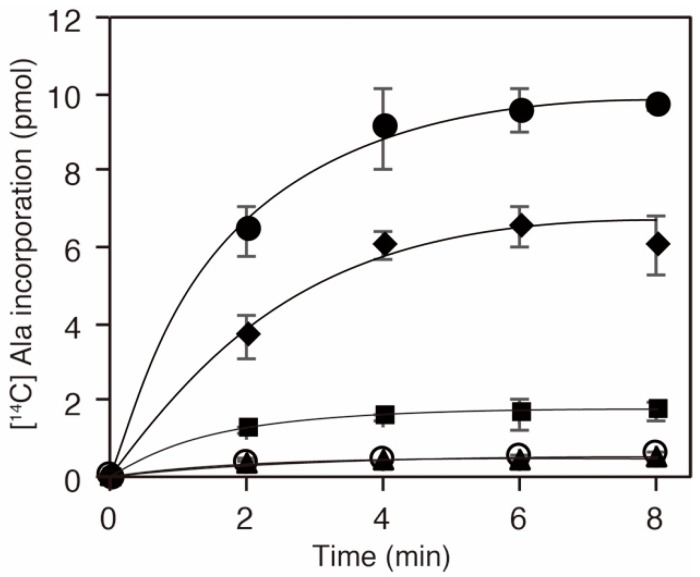
Alanylation activities of *E. coli* AlaRS K73Ac variants. In vitro transcribed *E. coli* tRNA^Ala^ was alanylated with AlaRS WT (filled circles), K73A (opened circles), K73Ac (NAM−; diamonds), K73Ac (NAM+; squares), and K73Ac (∆CobB; triangles). The data is shown as the average of three independent assays with the standard deviations.

Given that the K73 in AlaRS was important for the alanylation activity [29,30], we inferred that acetylation of the residue should influence the activity and consequently downregulate protein translation. To examine the activity of a series of AlaRS K73 variants, we performed alanylation assays in vitro (Figure 3). We confirmed that the K73A mutation abolished the alanylation activity of AlaRS, as reported previously [30]. AlaRS K73Ac (NAM−) showed decreased alanylation activity compared to the AlaRS WT. The alanylation activities of K73Ac (∆CobB) and K73Ac (NAM+) variants, which showed higher levels of acetylation than K73Ac (NAM−), were more impaired. Considering that the acetylation signals reflect only K73 acetylation, these results indicated that K73 acetylation inhibits the alanylation activity of AlaRS.

### 3.3. Circular Dichroism Spectrum of AlaRS K73Ac Variants

**Figure 4:**
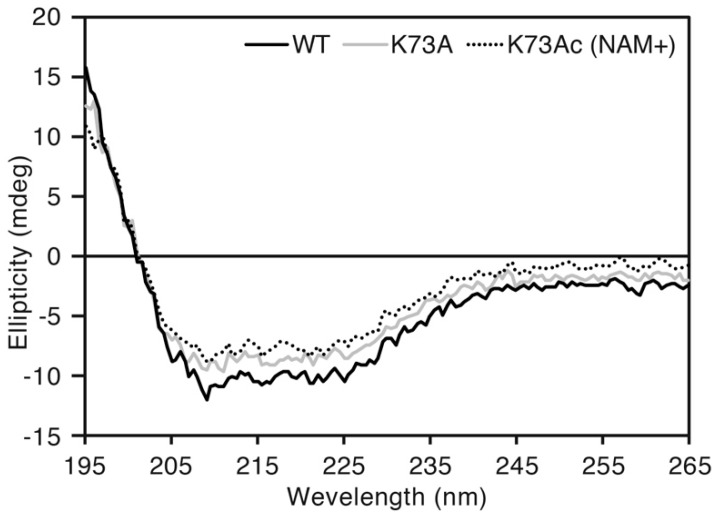
Circular dichroism (CD) spectral analysis. The black, gray, and dotted lines indicate AlaRS WT, K73A, and K73Ac (NAM+), respectively. Scanning was performed three times for each sample and the average was plotted.

To exclude the possibility of a structural defect caused by genetic incorporation of AcK, we compared CD spectra of AlaRS WT, K73A and K73Ac (NAM+). Almost the same spectral curves were observed among the three proteins (Figure 4). The proportion of secondary structures contained in those proteins was calculated by the CAPITO web server [34]. WT, K73A and K73Ac (NAM+) contained 21%, 13%, and 22% α-helix, and 22%, 24%, and 29% β-sheet, respectively. This suggested that incorporation of AcK did not impair the protein structure of AlaRS.

### 3.4. Escherichia coli Expresses Two CobB Isoforms with Each Promoter

**Figure 5:**
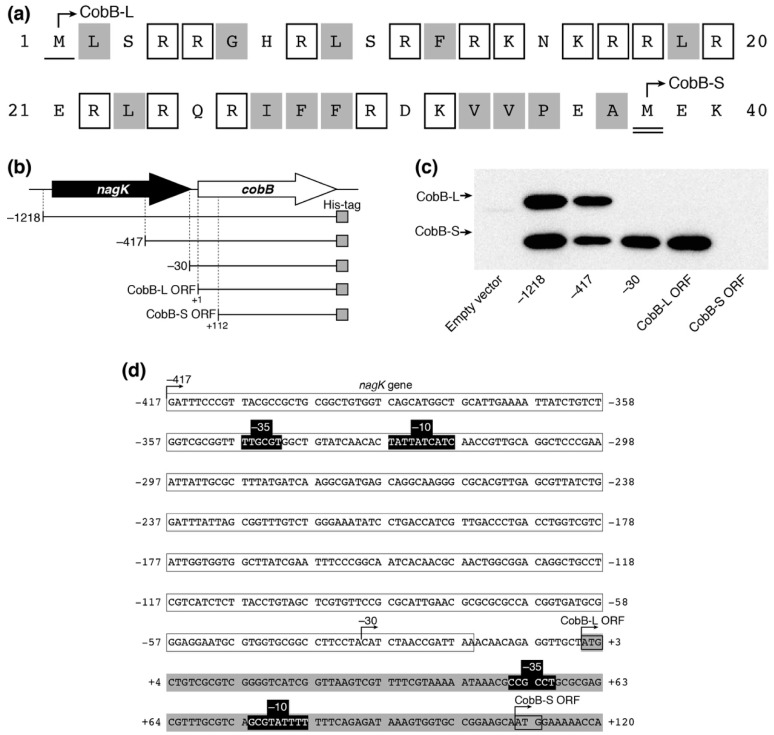
Expression of two CobB isoforms (CobB-L and CobB-S) in *E. coli*. (**a**) The primary sequence of N-terminal extension in CobB-L. The single and double underlines show the first methionine (Met) of CobB-L and CobB-S, respectively. The open and gray boxes indicate basic and hydrophobic amino acids, respectively (without Met). (**b**) Schematic representation of *cobB*-containing fragments in pACYC184P plasmid derivatives. (**c**) Expression of CobB isoforms in *E. coli*. Ten micrograms of lysate from *E. coli* DH10B harboring the pACYC184P derivatives was separated by 15% SDS-PAGE and analyzed by Western blotting using an anti-His-tag antibody to detect His-tagged CobB protein. (**d**) The nucleotide sequence of the *cobB* region. Open and gray boxes show the 3′-region of *nagK* and 5′-region of the *cobB* gene, respectively. The promoter containing −10 and −35 (black boxes) for CobB-L and CobB-S was predicted by BPROM of the Softberry web tool.

It has been reported that there are long and short isoforms of CobB (CobB-L and CobB-S, respectively) in *Salmonella enterica*, both of which are biologically active in the cell [35]. The existence of the two CobB isoforms has not been experimentally confirmed in *E. coli* yet. The *cobB* ORF in the public *E*. *coli* database encodes a 279-amino acid isoform (CobB-L), while a shorter 242-amino acid isoform (CobB-S) can be translated from the 112 nt-downstream second Met codon in the ORF. CobB-L contains a 37-amino acid N-terminal extension comprised of 15 basic and 13 hydrophobic residues, similar to CobB-L of *S*. *enterica* (Figure 5a). We expressed a C-terminal His-tagged CobB ORF with different upstream regions from pACYC184P plasmids in *E*. *coli* DH10B (Figure 5b). We detected two His-tagged protein bands by Western blotting, which corresponded to CobB-L and CobB-S from pACYC184P-1218-cobB and pACYC184P-417-cobB plasmids (Figure 5c). Meanwhile, CobB-S was only expressed from pACYC184P-30-cobB and pACYC184P-cobB-L. Neither form of CobB was detected with pACYC184P-cobB-S. These results indicated that the promoter for CobB-L expression existed between −417 and −30 within the *nagK* ORF and that for CobB-S expression existed between +1 and +111 within the *cobB* ORF. Using the online BPROM web tool (Softberry, Inc., Mount Kisco, NY, USA), we detected the candidates of the −10 and −35 consensus sequences between −347 and −319 for CobB-L and between +51 and +83 for CobB-S expression, respectively (Figure 5d). These results indicated that two isoforms of CobB-L and CobB-S exist in *E*. *coli* and they are directed from each promoter.

### 3.5. Deacetylation of K73 by CobB-S In Vitro

**Figure 6:**
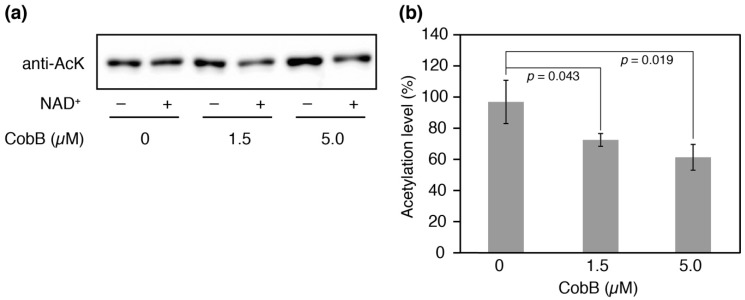
Deacetylation assay in vitro. (**a**) His-tagged AlaRS K73Ac (∆CobB) (0.5 µM) and indicated concentrations of His-tagged CobB-S were reacted at 37 °C for 12 h. After the reaction, the remaining acetylation in AlaRS was determined by Western blotting with an anti-AcK antibody. This experiment was repeated three times. (**b**) The proportion of acetylation levels was calculated from the ratio of band intensities with NAD^+^ to those without NAD^+^ (negative control). The data is shown as the result of three independent reactions with the standard deviations. The *p* values were determined by Student’s *t*-test.

We attempted to produce N-terminal His-tagged CobB isoform proteins for deacetylation assays in vitro, but CobB-L could not be successfully obtained as soluble protein after several attempts. CobB-S contains the conserved Sir2 catalytic core and has been shown to have the deacetylase activity for a histone H4 acetylated peptide [36]. We therefore performed an in vitro deacetylation assay with CobB-S. After incubation of the His-tagged CobB-S and AlaRS K73Ac (∆CobB) for 12 h, the residual acetylation was determined by Western blotting (Figure 6a). An NAD^+^-dependent decrease in acetylation was observed and it was enhanced by increasing concentrations of CobB-S; 27% and 39% decreases in acetylation were observed when 1.5 µM or 5.0 µM of CobB-S were used, respectively (Figure 6b). The deacetylase activity of CobB-S for AlaRS K73Ac seemed to be moderate, suggesting that CobB may require additional factors for full activity.

## 4. Discussion

Serine phosphorylation in glutamyl-tRNA synthetase [37], lysine succinylation in methionyl-tRNA synthetase (MetRS) [38], and lysine acetylation in LeuRS, ArgRS [22] and TyrRS [23] have been reported as meaningful PTMs toward the aaRS function in *E. coli*. These modifications, with the exception of lysine succinylation in MetRS, downregulate their tRNA synthetase activities. The lysine succinylation of MetRS decreases the discrimination of cognate tRNA under stress conditions [38]. In this study, we newly found K73 acetylation that downregulates the activity of *E. coli* AlaRS which belongs to class II aaRSs.

K73 in AlaRS is known to be an essential residue that interacts with the 3′-end of tRNA^Ala^ [29]. Substitution of this residue to glutamine, asparagine, alanine or glutamate significantly reduces the alanylation activity but not the alanine activation, suggesting that the positive charge of the lysine plays an important role in the interaction [30]. The recently described crystal structure of AlaRS with the cognate tRNA^Ala^ from *Archaeoglobus fulgidus* elucidated the recognition mechanism of tRNA^Ala^ by AlaRS [39]. Although *A. fulgidus* AlaRS lacks the corresponding lysine residue in the primary sequence, the cytidine-75 in the tRNA^Ala^ of the complex is located in the vicinity of K73 of the *E. coli* enzyme when its structure is superposed on the complex structure. Based on the above-mentioned evidence, our results suggest that downregulation of AlaRS activity by K73 acetylation is due to the loss of the positive charge in the lysine residue, resulting in interference of the interaction between AlaRS and tRNA^Ala^.

Our in vivo and in vitro studies indicate that the acetylation of K73 is reversed by CobB deacetylase. Systematic analysis to search for substrates of CobB has shown that CobB does not recognize a specific sequence, but tends to prefer aspartate (D), glutamate (E), alanine (A), glycine (G) and tyrosine (Y) on β-sheet or loop structures adjacent to an acetylated lysine [18]. The K73 exists in the AGGKHND sequence of the β-turn, which contains the favored amino acids of CobB (shown with underline). This observation supports our result that the acetylated K73 is a substrate of CobB. Our in vitro study showed the deacetylase activity of CobB-S for AlaRS K73Ac, but it seemed to be moderate. We consider the possibility that CobB-L may prefer to remove this acetylation or another factor like a chaperone might be required for the reaction.

We clarified the CobB-dependent deacetylation mechanism of K73 in AlaRS in vivo, while the mechanism of K73 acetylation remains elusive. The K73 acetylation has been detected at the stationary phase of growth without glucose [7,16] and at the early stationary phase when glucose is present [17]. In both cases, the intracellular level of acetyl-phosphate is likely increased by carbon overflow in the central metabolism pathway [16,40]. Thus, the acetylation of K73 is thought to be introduced by a non-enzymatic mechanism with acetyl-phosphate. This K73 acetylation has also been detected in *Vibrio parahaemolyticus*, mice, and humans [41,42]. The conserved K73 acetylation among different organisms may be of physiological significance, although it remains to be experimentally determined in other organisms.

Promoter analysis for CobB expression in *E. coli* was previously performed using an enhanced green fluorescent protein (EGFP)-based reporter system and real time quantitative PCR (RT-qPCR), showing that promoter activity for a *nagK*-*cobB* transcription was detected in the 5′-upstream region (−300 to +50) of *nagK* but no significant activity was detected in the upstream region (−400 to +151) of *cobB* [43]. Contrary to a previous report, we could find two promoter activities within the region of −417 to +111 of *cobB*: the one within *nagK* directs CobB-L expression and the other within *cobB* directs CobB-S expression (Figure 5d). These findings are similar to the case of *S. enterica*, in which the upstream and downstream promoters correspond to P2 and P3 promoters, respectively [35]. Since CobB-S contains the conserved catalytic core which is sufficient for the deacetylase activity [35,36], the extended N-terminal region composed of positively charged and hydrophobic amino acids may add an alternative function to CobB-L. It was proposed in *S. enterica* that the extended N-terminal region may be involved in interaction with other molecules such as nucleic acids and acetylated protein substrates, and cellular localization [35]. Our failure to purify N-terminal His-tagged CobB-L might be due to masking of the N-terminal His-tag by unknown interacting partners. Elucidation of the physiological significance of CobB-L and CobB-S is an intriguing challenge for the future studies.

As aaRSs are essential enzymes, severe inhibition of their activities causes growth retardation and cell death [44]. Therefore, it is thought that downregulation of AlaRS activity by K73 acetylation is likely transient and AlaRS should be rapidly reactivated by deacetylation when growth conditions are improved. Hence, our findings will provide insight into a new regulatory mechanism of translation by post-translational lysine acetylation of aaRSs. Future studies will shed light on the acetylation introduced into other aaRSs for a more comprehensive understanding of translation regulation.

## Appendix A

**Table A1:** Lysine acetylation sites in all aminoacyl-tRNA synthetases (aaRSs) from *E. coli*. The data was collected from the acetylomes performed by Kuhn et al. [16] and Schilling et al. [17].

aaRS	Acetyl Site	[16] Mascot Score	[17] Mascot Score		aaRS	Acetyl Site	[16] Mascot Score	[17] Mascot Score
AlaRS	55	45.4	58.7		GlnRS	160		71.2
	73	55.3	68.5			170	95.0	49.3
	278		85.2			195		93.0
	535	39.8	140.6			273	51.4	50.1
	591	53.8	71.2			399		41.8
	604		51.7			408	53.5	41.7
	647	70.8				419	57.9	
	721		59.0			515	34.3	65.4
	730	64.5	102.7		GluRS	102	41.1	
	743	57.6	97.9			215	50.9	65.0
	749	59.2	74.3			293		43.9
	763		53.9			345	107.9	77.7
	783	86.0	94.0			377	44.5	60.8
	819	54.8	101.2			423	70.4	
	873		51.8			459		44.3
ArgRS	112	53.4			GlyRS-α	3	38.1	42.5
	126	83.3	103.8			281	41.3	
	152	73.6			GlyRS-β	72	53.1	110.1
	198		50.6			86	72.4	73.5
	216	45.8	40.6			89		58.6
	295		44.5			112	75.6	
	306		54.3			141		54.1
	473		64.2			472		43.1
AsnRS	194		54.1			475	53.5	73.4
	199	51.9	42.3			592	55.1	99.6
	286	33.7	75.5			607	76.1	47.0
	294	53.1	67.1			624	43.5	59.1
	351	52.0	58.1			662	63.1	71.2
	357		66.1			674	52.4	46.3
	375		41.2		HisRS	53	51.5	72.0
AspRS	186	49.8	56.5			189		74.2
	283	65.2	80.6			370		47.3
	315		55.7			378	44.3	49.4
	332	57.1	54.9		IleRS	23	57.3	98.3
	353	58.5	78.1			112	61.9	82.3
	370	48.5				116	83.0	106.4
	399	35.8				183	56.6	66.6
	415	74.3	95.2			390		109.4
	513		73.1			398	90.2	96.7
CysRS	73	54.3	97.1			428	76.6	107.6
	76	50.8	90.9			436	57.1	58.1
	175	44.4				439	55.7	52.9
	282	53.4	59.4			605	49.1	
	310		48.4			619	108.3	104.3
						895		78.7
						913		50.0
						934	64.1	93.2
LeuRS	21	56.1	74.8		SerRS	17		54.2
	145	47.3				24	73.7	109.8
	182		54.7			29	49.6	102.6
	598	51.8	62.4			43	53.6	97.1
	619	53.2	81.4			77		42.2
	837		45.6			86		83.1
LysRS	82	112.9	109.9			115	68.7	
	114	88.0	123.2		ThrRS	122	87.8	95.8
	115	56.7	108.4			169	61.0	76.5
	156	57.9	132.8			200	52.3	
	335		91.8			226	61.8	76.3
LysRS	114	52.2	109.1			227	56.3	86.8
(Inducible)	115	52.0	109.2			286	56.9	
	156	119.0	116.1			314		68.6
	179	57.2	60.0			577	52.4	44.4
	322	76.7	95.4			599	52.6	58.6
	326		84.6			614	55.2	42.2
	335	37.9	103.6		TrpRS	173		47.2
MetRS	115		38.2			181	55.2	66.8
	121	48.6				214	57.6	76.6
	133	77.0	85.4			274		40.2
	143		38.2			307		46.9
	343	98.3	140.0		TyrRS	8	62.0	109.0
	403	50.0	54.8			85	76.7	131.3
	466		66.5			144	40.1	105.3
	498		85.4			235	55.2	62.5
	529	68.3	64.3			238	77.6	109.4
	597	68.1	124.5			416	55.1	
PheRS-α	34	47.1			ValRS	3		73.5
	75		71.0			178	52.7	45.9
	265	82.8				209		45.4
	324	31.9				248		35.2
PheRS-β	66	56.9				271	48.1	65.9
	104	70.9	86.8			291	52.9	101.4
	235		55.1			317	116.6	117.1
	371	66.6	85.0			334	70.9	68.6
	441	61.8	57.5			358		50.2
	496	42.1				593	65.9	96.3
	655	58.6	126.3			600		79.6
	780		42.9			633		41.2
ProRS	34	76.2	45.0			644	53.6	56.8
	126	78.2	91.0			861	76.9	110.1
	420		80.7			898		70.4
	495	41.8				909	108.8	104.5
	554	56.7	57.0			926	44.9	76.2
	568		78.9			930	48.3	80.0

**Table A2:** List of primers for PCR.

Name	Sequence	Purpose
pT5C-Ec-alaS-F	AGAGGAGAAATTACATATGAGCAAGAGCACCGCTG	Construction of pT5C-Ec-alaS
pT5C-Ec-alaS-R	TGGTGGTGGTGGTGCTCGAGTGCGGCTTGCAATTTC	
Ec-alaS-K74A-F	CTGCGTGCGTGCGGGTGGTGCGCACAACGACCTGGAAAAC	Construction of pT5C-Ec-alaS-K74A
Ec-alaS-K74A-R	GTTTTCCAGGTCGTTGTGCGCACCACCCGCACGCACGCAG	
Ec-alaS-K74amb-F	CTGCGTGCGTGCGGGTGGTTAGCACAACGACCTGGAAAAC	Construction of pT5C-Ec-alaS-K74amb
Ec-alaS-K74amb-R	GTTTTCCAGGTCGTTGTGCTAACCACCCGCACGCACGCAG	
pTECH-1960-Nhe-F	GGAAGCTAGCCTGTTGCCCGTCTCACTGGTGAAAAG	Construction of pKTS-AcKRS1-PylT
pTECH-1683-Nhe-R	GGAAGCTAGCCTGGCGAAAGGGGGATGTGCTG	
cobB-Nde-F	GTCACATATGCTGTCGCGTCGGGGTCATC	Construction of pET15b-cobB-L and pET15b-cobB-S
cobB-S-Nde-F	TGCCATATGGAAAAACCAAGAGTACTCGTACTGAC	
cobB-Bam-R	GACTGGATCCTTAGGCAATGCTTCCCGCTTTTAATC	
Ec-cobB-KO-F	GTGGTGCGGCCTTCCTACATCTAACCGATTAAACAACAGAGGTTGCTATGATTCCGGGGATCCGTCGACC	Disruption of *cobB* gene
Ec-cobB-KO-R	CCCCTTGCAGGCCTGATAAGCGTAGTGCATCAGGCAATGCTTCCCGCTTTTGTAGGCTGGAGCTGCTTCG	
cobB-1218-BglII-F	AGCAGATCTGTTCGGCAGCCTGTGTGGTGTG	Construction of pACYC184P-1218-cobB, -417-cobB, -30-cobB, -cobB-L, and -cobB-S
cobB-417-BglII-F	AGCAGATCTGATTTCCCGTTACGCCGCTG	
cobB-30-BglII-F	AGCAGATCTCATCTAACCGATTAAACAACAGAGGTTGC	
cobB-L-BglII-F	CAGAGATCTATGCTGTCGCGTCGGGGTC	
cobB-S-BglII-F	CAGAGATCTATGGAAAAACCAAGAGTACTCGTACTG	
cobB-XhoI-R	GCTCTCGAGGGCAATGCTTCCCGCTTTTAATCC	

**Table A3:** List of plasmids in this study. nts, nucleotides.

Plasmids	Description
pT5C-Ec-alaS	AlaRS wild type expression
pT5C-Ec-alaS-K74A	AlaRS K73A expression
pT5C-Ec-alaS-K74amb	AlaRS K73Ac expression
pKTS-AcKRS1-PylT	AcK incorporation system
pACYC184P	Empty vector for CobB expression analysis
pACYC184P-1218-cobB	*cobB* gene with 1218 nts upstream sequence
pACYC184P-417-cobB	*cobB* gene with 417 nts upstream sequence
pACYC184P-30-cobB	*cobB* gene with 30 nts upstream sequence
pACYC184P-cobB-L	*cobB* gene (long length) ORF
pACYC184P-cobB-S	*cobB* gene (short form) ORF
pET15b-cobB-L	CobB-L expression
pET15b-cobB-S	CobB-S expression

**Table A4:** *E. coli* strains in this study.

*E. coli* Strains	Genotype	Source
DH10B	*F*^−^*, mcrA, Δ(mrr*-*hsdRMS*-*mcrBC), φ80lacZΔM15, ΔlacX74, recA1, endA1, araD139, Δ(ara*-*leu)7697, galU, galK, λ*^−^*, rpsL(Str^R^), nupG*	Invitrogen
BL21(DE3)	*F^−^, dcm, ompT, hsdS(r_B_^−^ m_B_^−^), gal, λ(DE3)*	Nippon Gene
∆CobB	DH10B *cobB::frt*	This study

## References

[1] Cain J.A., Solis N., Cordwell S.J. (2014). Beyond gene expression: The impact of protein post-translational modifications in bacteria. J. Proteom..

[2] Ouidir T., Kentache T., Hardouin J. (2016). Protein lysine acetylation in bacteria: Current state of the art. Proteomics.

[3] Liu L., Wang G., Song L., Lv B., Liang W. (2016). Acetylome analysis reveals the involvement of lysine acetylation in biosynthesis of antibiotics in *Bacillus amyloliquefaciens*. Sci. Rep..

[4] Liu J., Wang Q., Jiang X., Yang H., Zhao D., Han J., Luo Y., Xiang H. (2017). Systematic analysis of lysine acetylation in the halophilic archaeon *Haloferax mediterr*. J. Proteom. Res..

[5] Kosono S., Tamura M., Suzuki S., Kawamura Y., Yoshida A., Nishiyama M., Yoshida M. (2015). Changes in the acetylome and succinylome of *Bacillus subtilis* in response to carbon source. PLoS ONE.

[6] Kentache T., Jouenne T., De E., Hardouin J. (2016). Proteomic characterization of *N*_α_- and *N_ε_*-acetylation in *Acinetobacter baumannii*. J. Proteom..

[7] Baeza J., Dowell J.A., Smallegan M.J., Fan J., Amador-Noguez D., Khan Z., Denu J.M. (2014). Stoichiometry of site-specific lysine acetylation in an entire proteome. J. Biol. Chem..

[8] Hentchel K.L., Escalante-Semerena J.C. (2015). Acylation of biomolecules in prokaryotes: A widespread strategy for the control of biological function and metabolic stress. Microbiol. Mol. Biol. Rev..

[9] Zhang Q.F., Gu J., Gong P., Wang X.D., Tu S., Bi L.J., Yu Z.N., Zhang Z.P., Cui Z.Q., Wei H.P. (2013). Reversibly acetylated lysine residues play important roles in the enzymatic activity of *Escherichia coli N*-hydroxyarylamine *O*-acetyltransferase. FEBS J..

[10] Song L., Wang G., Malhotra A., Deutscher M.P., Liang W. (2016). Reversible acetylation on Lys501 regulates the activity of RNase II. Nucleic Acids Res..

[11] Thao S., Chen C.S., Zhu H., Escalante-Semerena J.C. (2010). *N^ε^*-lysine acetylation of a bacterial transcription factor inhibits its DNA-binding activity. PLoS ONE.

[12] Liang W., Malhotra A., Deutscher M.P. (2011). Acetylation regulates the stability of a bacterial protein: Growth stage-dependent modification of RNase R. Mol. Cell.

[13] Ren J., Sang Y., Tan Y., Tao J., Ni J., Liu S., Fan X., Zhao W., Lu J., Wu W. (2016). Acetylation of lysine 201 inhibits the DNA-binding ability of PhoP to regulate *Salmonella* virulence. PLoS Pathog.

[14] Ramakrishnan R., Schuster M., Bourret R.B. (1998). Acetylation at Lys-92 enhances signaling by the chemotaxis response regulator protein CheY. Proc. Natl. Acad. Sci. USA.

[15] Weinert B.T., Iesmantavicius V., Wagner S.A., Scholz C., Gummesson B., Beli P., Nystrom T., Choudhary C. (2013). Acetyl-phosphate is a critical determinant of lysine acetylation in *E. coli*. Mol. Cell.

[16] Kuhn M.L., Zemaitaitis B., Hu L.I., Sahu A., Sorensen D., Minasov G., Lima B.P., Scholle M., Mrksich M., Anderson W.F. (2014). Structural, kinetic and proteomic characterization of acetyl phosphate-dependent bacterial protein acetylation. PLoS ONE.

[17] Schilling B., Christensen D., Davis R., Sahu A.K., Hu L.I., Walker-Peddakotla A., Sorensen D.J., Zemaitaitis B., Gibson B.W., Wolfe A.J. (2015). Protein acetylation dynamics in response to carbon overflow in *Escherichia coli*. Mol. Microbiol..

[18] AbouElfetouh A., Kuhn M.L., Hu L.I., Scholle M.D., Sorensen D.J., Sahu A.K., Becher D., Antelmann H., Mrksich M., Anderson W.F. (2015). The *E. coli* sirtuin CobB shows no preference for enzymatic and nonenzymatic lysine acetylation substrate sites. Microbiologyopen.

[19] Zhang J., Sprung R., Pei J., Tan X., Kim S., Zhu H., Liu C.F., Grishin N.V., Zhao Y. (2009). Lysine acetylation is a highly abundant and evolutionarily conserved modification in *Escherichia coli*. Mol. Cell. Proteom..

[20] Zhang K., Zheng S., Yang J.S., Chen Y., Cheng Z. (2013). Comprehensive profiling of protein lysine acetylation in *Escherichia coli*. J. Proteom. Res..

[21] Moras D. (1992). Structural and functional relationships between aminoacyl-tRNA synthetases. Trends Biochem. Sci..

[22] Ye Q., Ji Q.Q., Yan W., Yang F., Wang E.D. (2017). Acetylation of lysine ϵ-amino groups regulates aminoacyl-tRNA synthetase activity in *Escherichia coli*. J. Biol. Chem..

[23] Venkat S., Gregory C., Gan Q., Fan C. (2017). Biochemical characterization of the lysine acetylation of tyrosyl-tRNA synthetase in *Escherichia coli*. Chembiochem.

[24] Putney S.D., Sauer R.T., Schimmel P.R. (1981). Purification and properties of alanine tRNA synthetase from *Escherichia coli* A tetramer of identical subunits. J. Biol. Chem..

[25] Umehara T., Kim J., Lee S., Guo L.T., Söll D., Park H.S. (2012). *N*-Acetyl lysyl-tRNA synthetases evolved by a CcdB-based selection possess *N*-acetyl lysine specificity in vitro and in vivo. FEBS Lett..

[26] Baba T., Ara T., Hasegawa M., Takai Y., Okumura Y., Baba M., Datsenko K.A., Tomita M., Wanner B.L., Mori H. (2006). Construction of *Escherichia coli* K-12 in-frame, single-gene knockout mutants: The Keio collection. Mol. Syst. Biol..

[27] Datsenko K.A., Wanner B.L. (2000). One-step inactivation of chromosomal genes in *Escherichia coli* K-12 using PCR products. Proc. Natl. Acad. Sci. USA.

[28] Swairjo M.A., Otero F.J., Yang X.L., Lovato M.A., Skene R.J., McRee D.E., Ribas de Pouplana L., Schimmel P. (2004). Alanyl-tRNA synthetase crystal structure and design for acceptor-stem recognition. Mol. Cell.

[29] Hill K., Schimmel P. (1989). Evidence that the 3′ end of a tRNA binds to a site in the adenylate synthesis domain of an aminoacyl-tRNA synthetase. Biochemistry.

[30] Filley S.J., Hill K.A. (1993). Amino acid substitutions at position 73 in motif 2 of *Escherichia coli* alanyl-tRNA synthetase. Arch. Biochem. Biophys..

[31] Dumas A., Lercher L., Spicer C.D., Davis B.G. (2015). Designing logical codon reassignment—Expanding the chemistry in biology. Chem. Sci..

[32] Larkin M.A., Blackshields G., Brown N.P., Chenna R., McGettigan P.A., McWilliam H., Valentin F., Wallace I.M., Wilm A., Lopez R. (2007). Clustal W and Clustal X version 2.0. Bioinformatics.

[33] Robert X., Gouet P. (2014). Deciphering key features in protein structures with the new ENDscript server. Nucleic Acids Res..

[34] Wiedemann C., Bellstedt P., Gorlach M. (2013). CAPITO—A web server-based analysis and plotting tool for circular dichroism data. Bioinformatics.

[35] Tucker A.C., Escalante-Semerena J.C. (2010). Biologically active isoforms of CobB sirtuin deacetylase in *Salmonella enterica* and *Erwinia amylovora*. J. Bacteriol..

[36] Zhao K., Chai X., Marmorstein R. (2004). Structure and substrate binding properties of cobB, a Sir2 homolog protein deacetylase from *Escherichia coli*. J. Mol. Biol..

[37] Germain E., Castro-Roa D., Zenkin N., Gerdes K. (2013). Molecular mechanism of bacterial persistence by HipA. Mol. Cell..

[38] Schwartz M.H., Waldbauer J.R., Zhang L., Pan T. (2016). Global tRNA misacylation induced by anaerobiosis and antibiotic exposure broadly increases stress resistance in *Escherichia coli*. Nucleic Acids Res..

[39] Naganuma M., Sekine S., Chong Y.E., Guo M., Yang X.L., Gamper H., Hou Y.M., Schimmel P., Yokoyama S. (2014). The selective tRNA aminoacylation mechanism based on a single G•U pair. Nature.

[40] Klein A.H., Shulla A., Reimann S.A., Keating D.H., Wolfe A.J. (2007). The intracellular concentration of acetyl phosphate in *Escherichia coli* is sufficient for direct phosphorylation of two-component response regulators. J. Bacteriol..

[41] Pan J., Ye Z., Cheng Z., Peng X., Wen L., Zhao F. (2014). Systematic analysis of the lysine acetylome in *Vibrio parahemolyticus*. J. Proteom. Res..

[42] Hornbeck P.V., Kornhauser J.M., Tkachev S., Zhang B., Skrzypek E., Murray B., Latham V., Sullivan M. (2012). PhosphoSitePlus: A comprehensive resource for investigating the structure and function of experimentally determined post-translational modifications in man and mouse. Nucleic Acids Res..

[43] Castano-Cerezo S., Bernal V., Blanco-Catala J., Iborra J.L., Canovas M. (2011). cAMP-CRP co-ordinates the expression of the protein acetylation pathway with central metabolism in *Escherichia coli*. Mol. Microbiol..

[44] Ho J.M., Bakkalbasi E., Söll D., Miller C.A. (2018). Drugging tRNA aminoacylation. RNA Biol..

